# Stuck on the Last: The Last-Presented Benefit as an Index of Attentional Refreshing in Adolescents

**DOI:** 10.3390/jintelligence11010004

**Published:** 2022-12-23

**Authors:** Beatrice Valentini, Evie Vergauwe

**Affiliations:** Faculty of Psychology and Science of Education, University of Geneva, Bureau 5158, 40 Boulevard Pont d’Arve, 1205 Genève, Switzerland

**Keywords:** working memory, attention, attentional refreshing, last-presented benefit

## Abstract

Working memory is a limited-capacity system responsible for maintaining information that is known to dramatically develop throughout childhood and adolescence. Different maintenance mechanisms are proposed to support working memory development, among which is attentional refreshing. Attentional refreshing is assumed to improve the accessibility of working-memory representations by cycling attention from one mental representation to the other, serially. It has been suggested that the efficiency of refreshing increases between the ages of 7 and 14 years old, thereby supporting working memory development. Yet, there is not much research about refreshing in adolescence. Here, we investigate the occurrence of refreshing in 15-year-olds by using a recently-developed index, i.e., the last-presented benefit. Adolescents had to remember a list of four letters and judge whether a subsequent probe letter was present or not in the list. Reaction times to the probe were used to assess the spontaneous occurrence of refreshing. We found that, unlike young adults, 15-year-olds showed consistent speeded responses to probes matching the last-presented memory item, indicating that, in this task, adolescents did not refocus their attention away from the last memory item to initiate refreshing. Implications for working memory functioning and development are discussed.

## 1. Introduction

Working memory is the limited-capacity cognitive system that is responsible for maintaining information that it is no longer perceptually available, and is related to a range of high-level cognitive abilities including fluid intelligence (Ackerman et al. 2002; Burgess et al. 2011; Conway et al. 2003; Engel de Abreu et al. 2010; Fukuda et al. 2010; Kane et al. 2005; Kyllonen and Christal 1990). Working memory develops dramatically during childhood with a peak at around 20 years old (Brockmole and Logie 2013; Mitchell et al. 2000; Sander et al. 2011). However, there are at least two main aspects of working memory development that are still to be clarified: (1) At what point in the development can working memory capacity be considered adult-like? (2) What is the role of working memory maintenance mechanisms in achieving adult-like working memory performance? Below, we address each of these aspects, highlighting some of the main open questions related to these aspects, and explain how the current study aims to provide relevant data that speak to these questions.

### 1.1. When Does Working Memory Performance Reach Adult-like Levels?

Many studies reported that working memory capacity reaches adult levels at the age of 10–11 years (e.g., Logie and Pearson 1997; Riggs et al. 2006; Wilson et al. 1987) or during mid-adolescence (Gathercole et al. 2004; Pascual-Leone 1970). This is counterintuitive because large-scale lifespan studies show that working memory peaks around 20 years old (e.g., Brockmole and Logie 2013). Thus, it is unclear whether working memory development continues during adolescence or whether it reaches adult levels before early adolescence.

It is possible that studies estimating adult-level working memory capacity during mid- to late-childhood might have reached a premature conclusion. Indeed, some of these studies lack of a direct comparison between children and adults’ performance. For example, Riggs et al. (2006) do not test adults. Instead, they just report the capacity estimates of 10-year-olds (3.83 items) and judge them as comparable to estimates found in adults (i.e., 3 or 4 items, Luck and Vogel 1997; Vogel et al. 2001). Yet, the paradigm by Riggs et al. (2006) was adapted from the adult literature to be child-friendly (i.e., with slower encoding times than the adult version), so it is possible that the same adapted task would produce higher estimates in adults. 

Relatedly, the evidence in favor of adult-like performance levels in 10-year-olds might be linked to the paradoxical finding that infants seem to have higher working memory capacity than young children (see Cowan 2016; Forsberg et al. 2022). Indeed, infants of 9–12 months of age have been found to display adult-like capacity (around 3 items, Feigenson and Carey 2005; Kibbe and Leslie 2013; Zosh et al. 2011). However, infant and child procedures are not equivalent; infants’ procedures are much simpler than children’s ones. Thus, infants’ procedures overestimate infants’ capacity compared to children’s working memory capacity. Similarly, working memory tasks used in children are usually adapted from the adults’ tasks by using more familiar memoranda and by increasing encoding time, which may overestimate children’s capacity without a controlled comparison with adults. In fact, the direct comparison of children’ performance with adults’ might result in incorrect conclusions when not supported by data gathered in participants between the two age extremes, i.e., adolescents.

Finally, some papers compare children’s performance to adults who might not be at peak performance anymore. For example, Wilson et al. (1987) tested 5-, 7-, 11- and 35-year-olds and found comparable estimates between 11- and 35-year-olds. Yet, working memory in 35-year-olds might already be declining (Brockmole and Logie 2013; Jenkins et al. 1999; Swanson 1999, 2017), potentially resulting in a suboptimal age comparison.

Overall, not many studies focused on the development of working memory capacity between 11 years of age and early adulthood (but see Gathercole et al. 2004; Isbell et al. 2015; Kwon et al. 2002; Luciana et al. 2005; Luna et al. 2004; Sander et al. 2011), making it difficult to draw strong conclusions on when working memory performance becomes adult-like. Yet, we argue that studying this age group might be extremely valuable in a theoretical sense. In fact, it is a privileged stage before the peak of working memory development, so testing this age group might allow us to capture the onset and ongoing maturation of specific working memory mechanisms and strategies that would support the peak performance observed in young adults. In particular, it is currently unclear whether adolescents can spontaneously use maintenance mechanisms to achieve adult-like performance. This is one of the open questions that the current study will address, with a specific focus on an attention-based mechanism called refreshing^1^.

### 1.2. The Role of Maintenance Mechanisms in Achieving Adult-like Working Memory Performance

Two of the main maintenance mechanisms that have been proposed to support working memory performance in adults (e.g., Camos et al. 2009) and working memory development in children (e.g., Oftinger and Camos 2018) are articulatory rehearsal and attentional refreshing. Articulatory rehearsal refers to a domain-specific mechanism that supports the maintenance of verbal information by verbally repeating the to-be-remembered information, either overtly or covertly (Baddeley 1986, 2012; Camos et al. 2009). It has been proposed that children start to spontaneously use articulatory rehearsal from the age of 7 years (Baddeley et al. 1998; Flavell et al. 1966; Gathercole 1998; Gathercole et al. 1994; Gathercole and Adams 1993; Henry 1991; Hitch et al. 1989; Hulme et al. 1984; Keeney et al. 1967; but see (Elliott et al. 2021). Moreover, it is often assumed that the efficiency of articulatory rehearsal increases with age during childhood, supporting the development of working memory (e.g., Gathercole et al. 2004; Hulme et al. 1984; Tam et al. 2010). In particular, Hulme et al. (1984) plotted the articulation rate as a function of the memory span for four different age groups ranging from 4-year-olds to young adults. Their rationale was that articulation rate is equivalent to the articulatory rehearsal rate; thus, they were expecting (1) articulation rate to increase as a function of age, and (2) a similar relation between articulation rate and memory span in each one of the age groups. This is exactly what they found, and the authors concluded that the development of articulatory rehearsal with age is indeed what drives working memory development. However, a study by Cowan et al. (2011) seems to show that, in a condition of articulatory suppression (i.e., when the use of articulatory rehearsal to maintain information in working memory is minimized by requiring participants to utter irrelevant syllables in a continuous fashion), young adults still outperform 12-year-olds, showing that some other mechanisms must support adult-like performance, beyond articulatory rehearsal (see also Dempster 1981).

Another popular maintenance mechanism that has been proposed to support working memory development is attentional refreshing. Refreshing is a maintenance mechanism that is assumed to reactivate information in working memory through the use of attention (Barrouillet et al. 2009; Camos et al. 2018; Raye et al. 2007). Refreshing is assumed to emerge around the age of 7 years old and its efficiency is assumed to increase over time until around 14 years old (Barrouillet et al. 2009). Refreshing has been extensively studied in adults (e.g., Barrouillet et al. 2011; Lemaire et al. 2018; Loaiza and Souza 2018; Oberauer and Lewandowsky 2011; Raye et al. 2007, 2008; Rey et al. 2018; Souza et al. 2015; Vergauwe et al. 2014; Vergauwe and Langerock 2017) and, albeit to a lower degree, in children (e.g., Barrouillet et al. 2009; Oftinger and Camos 2015, 2017, 2018; Shimi and Scerif 2015, 2017, 2022; Tam et al. 2010; Vergauwe et al. 2021). However, evidence about attentional refreshing in adolescence is particularly scarce. In fact, to our knowledge, only two experiments have ever tested attentional refreshing in adolescents, i.e., Experiment 1 and 2 in Barrouillet et al. (2009) testing 14-year-olds. 

Additional evidence is needed to verify whether spontaneous attentional refreshing occurs in adolescents, and whether its functioning is equivalent between adolescents and young adults. Indeed, a set of recent studies used a newly-developed index of spontaneous refreshing and found that, in a very simple working memory task, there was evidence for the spontaneous use of refreshing in young adults (Vergauwe and Langerock 2017). However, using the same task in 9- and 12-year-old children did not show any evidence for the spontaneous use of refreshing in middle childhood (Vergauwe et al. 2021). The observation of spontaneous refreshing in young adults but not in 12-year-olds raises the question of whether adolescents use spontaneous refreshing in highly similar task settings. To answer this question, it is necessary to investigate spontaneous attentional refreshing in 15-year-old adolescents. Here, we proposed to do so using the last-presented benefit, an independent, direct index that has been used previously to detect refreshing both in adults (Valentini et al. 2022; Vergauwe and Langerock 2017) and in children (Vergauwe et al. 2021).

### 1.3. The Last-Presented Benefit

Vergauwe and Langerock (2017) proposed a new way to detect the occurrence of spontaneous refreshing by analyzing the reaction times in an item-recognition task (Sternberg 1966). Specifically, they presented to young adults a list of four to-be-maintained letters, followed by a probe letter to be judged as present in or absent from the memory list. Participants had to make their judgment as quickly as possible while minimizing errors. Reaction times to probes matching the last-presented item were compared with reaction times to probes matching the other list items. The authors reasoned that, if participants do not use attentional refreshing, their attentional focus should remain on the last-encoded memory item (i.e., the last-presented item); thus, a last-presented benefit should be observed whereby reaction times are faster for probes matching the last-presented item relative to probes matching any of the other memory items. In contrast, if participants spontaneously use attentional refreshing, their attentional focus should switch away from the last-encoded memory item in order to cyclically reactivate the other to-be-remembered items represented in working memory. As a result, reaction times should no longer be particularly fast for probes matching the last-presented memory item. Thus, the observation of a last-presented benefit is assumed to reflect the focus of attention lingering on the last-presented memory item and is not compatible with the occurrence of attentional refreshing which, by definition, requires the focus of attention to switch to other memory items to be initiated.

In adults, the results showed strong evidence for a last-presented benefit when the memory list was presented at a fast pace (i.e., 1 letter every 350 ms or every 500 ms) such that no time was available to refresh (Vergauwe and Langerock 2017). Yet, when the memory list was presented at a slower pace (i.e., 1 letter every 1 s), the last-presented benefit disappeared. The authors suggested that the disappearance of the last-presented benefit was an index of refreshing; when young adults had enough time to refresh during list presentation, their attention switched away from the last-presented item to refresh the to-be-remembered letters (Vergauwe and Langerock 2017). Next, the authors adapted this paradigm to study spontaneous attentional refreshing in school-aged children (Vergauwe et al. 2021). However, they observed that, in 9- and 12-year-olds, the last-presented benefit did not disappear, even when the memory list was presentated at a very slow pace (i.e., 1 letter every 1.5 s or every 2.5 s, see Table 1). This suggests that children did not use spontaneous refreshing, even at 12 years old, in the same task in which it had been shown that young adults do. It raises the question of when the disappearance of the last-presented benefit as a function of a slow-enough item presentation might start to occur. The present study aimed at making a first step towards answer this question by testing the presence or absence of the last-presented benefit in 15-year-olds with a presentation rate of 1 item per 1500 ms (i.e., a rate that is slower than the rate at which adults were observed to use refreshing, i.e., 1 item per 1000 ms).

It should be noted that the current study provides a first, and rather exploratory, step towards understanding refreshing during adolescence. To do so, we decided to test the occurrence of refreshing in 15-year-olds in a task situation in which it is reasonable to assume that there is enough free time for refreshing to take place. To detect refreshing, we used the last-presented benefit. Several elements informed our decision to test 15-year-olds (as opposed to, for example, 13-year-olds or 17-year-olds). First, the age of 15 years old falls roughly in the middle of the ages of the groups that have been tested using the last-presented benefit in previous studies (12-year-olds and young adults). Second, 15-year-olds are rather close in age to 14-year-olds, which is an age group that is assumed to be able to use attentional refreshing spontaneously and efficiently (Barrouillet et al. 2009). Finally, we also chose 15-year-olds out of recruitment convenience. In fact, in the Geneva school system, 15-year-olds are attending the last year of obligatory school which presents more diverse classes compared to classes in post-compulsory education.

### 1.4. The Present Study

The present study aims at testing the spontaneous occurrence of attentional refreshing in 15-year-olds using the last-presented benefit index.

To do so, we adapted the paradigm used by Vergauwe and colleagues (Vergauwe and Langerock 2017; Vergauwe et al. 2021) to an adolescent sample. We decided to limit our study to only a slow presentation rate because the presence of the last-presented benefit is robust at fast item presentation rates in both children and young adults (Valentini et al. 2022; Vergauwe and Langerock 2017; Vergauwe et al. 2021; see Table 1 for an overview). We decided to test whether using a slow presentation rate in adolescents, the last-presented benefit will be present (indicating that no refreshing occurred, like in 9- and 12- year-olds) or absent (indicating that refreshing occurred, like in adults). Accordingly, we presented the to-be-remembered letters at a rate of 1500 ms/item, which has also been used in children as young as 9 years old (Vergauwe et al. 2021), and which is 1.5 times slower than the slow rate used in adults (1000 ms/item, in Vergauwe and Langerock 2017).

If adolescents use working memory similarly to adults, then they should spontaneously use refreshing in this specific task setting for which it has been shown adults do (Vergauwe and Langerock 2017). In this case, like in adults, there should not be a last-presented benefit. However, in the case that there is still an important evolution of the maintenance mechanisms in working memory during adolescence, it is possible that, as with 9- and 12-year-olds, there is still evidence for a last-presented benefit, indicating that refreshing is not spontaneously used by 15-year-olds.

## 2. Methods

### 2.1. Participants

Thirty-seven adolescents (M age = 180.97 months, SD = 4.93, 19 girls) were recruited for this study using the established procedure to gain authorization to test children enrolled in the public schools in Geneva. Similar sample sizes were reported in previously published studies using item recognition tasks in our lab in both adults (e.g., Vergauwe and Langerock 2017) and children (e.g., Vergauwe et al. 2021). Prior to testing, written consent was required from the parents/guardians of the participants. All participants had correct-to-normal vision. Information regarding possible disorders was not available for this study and thus we did not apply any exclusions related to this.

### 2.2. Exclusion Criteria

Like in the previous studies run in our lab (Vergauwe et al. 2021; Vergauwe and Langerock 2017), the data of the participants displaying an average accuracy across the different probes types below 55% were discarded. This exclusion criterion led to the exclusion of one participant. Moreover, one participant was excluded due to technical problems (i.e., the program stopped in the middle of the task). This means that the total sample of analyzed data included the data from 35 adolescents.

### 2.3. Material and Procedure

**Figure 1:**
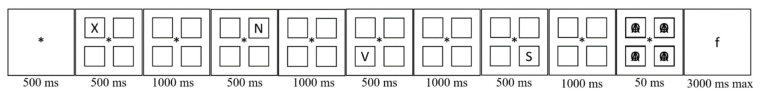
Illustration of the events on a single trial.

The task was administered using E-Prime3 (Psychology Software Tools). The program and the materials are all available at https://osf.io/ay87u/ (accessed on 21 September 2022). Participants were tested in a quiet room in their school in groups of approximately 7 participants at a time. The task duration was around 25–30 min. Each participant sat at a comfortable distance from the computer. Participants were asked to memorize a list of four letters presented sequentially, chosen randomly without replacement from a set of 18 consonants (all except W, Y, and Z), and to judge whether a following probe letter was presented or not in the list (see Figure 1).

Each letter was presented in one of the four boxes shown on the screen, namely two in the upper part of the screen and two in the lower part (see Figure 1). Each to-be-remembered letter was presented in the center of each box, in Courier New Font, 32 points, in upper case. Each box was 5.7 cm by 3.2 cm with a thin, black border. The boxes were arranged around the center with a horizontal separation of 1.1 cm, and a vertical separation of 1.2 cm.

Each series began with a fixation cross centrally displayed for 500 ms. Afterwards, the four letters to be remembered were presented sequentially in the four boxes: namely, the first to-be-remembered letter was shown in the upper-left box, the second letter in the upper-right box, the third one in the lower-left box and the fourth one in the lower-right box. Each letter was displayed for 500 ms, followed by a pattern of the four empty boxes for 1000 ms. At the end of the presentation phase, each box was filled for 50 ms with a mask created by the superposition of the letters A, O and I in Courier New Font, 32 points. 

The probe was displayed immediately after the mask, in the center of the screen, and it consisted of a letter displayed in lower case. The probe corresponded in 1/3 of trials to the last-presented letter (last-presented probe), in 1/3 of trials to any of the presented letters but the last one (not-last-presented probe), and in 1/3 of trials to a random new letter (new probe). This distribution was chosen to optimize the amount of data points per cell, and it was used in previous studies using this paradigm in adults (Vergauwe and Langerock 2017) and in children (Vergauwe et al. 2021). In order to judge the probes, the participant had to press the “L” key on the right of the keyboard (marked with a green sticker) when the probe was present in the series, and the “A” key on the left of the keyboard (marked with a red sticker) when the probe was absent. The probe disappeared upon the participant’s response. In the absence of response, the probe was presented on the screen for 3 s. 

Participants were instructed to judge the probe as fast as possible without making any error. Following their response, participants had to press the space bar in order to start the next trial. A phase of training and 6 practice trials (with explicit feedback on whether the answer was correct, incorrect or absent) preceded the 105 experimental trials (35 per probe type). After 45 experimental trials, participants were shown a screen informing them on their progress^2^.

### 2.4. Statistical Analysis

Following the previous literature on the last-presented benefit (see Table 1), the evidence in favor and against a last-presented benefit were tested directly using a paired, one-sided Bayesian *t*-test (see also Rouder et al. 2009). Bayesian *t*-tests compute the Bayes factor, which is used to quantify how much one model (e.g., the null) is more likely than the other (e.g., the alternative). Specifically, in the present study, the alternative hypothesis is that there is a difference in the reaction times to the probes, such that reaction times to probes matching the last-presented item are faster than reaction times to probes matching not-last-presented items (i.e., last-presented benefit). All analyses were run in R (BayesFactor package; Morey and Rouder 2015), with default settings. 

## 3. Results

Participants had high rates of correct responses to the probes across all probe type conditions (93% for last-presented, 88% for new, 80% for not-last-presented)^3^. Reaction time (RT) analyses only included correct responses. All materials and code can be found in https://osf.io/ay87u/. Abnormally fast trials (i.e., with RT < 150 ms) were removed from the analysis (see Uittenhove and Vergauwe 2019).

**Figure 2:**
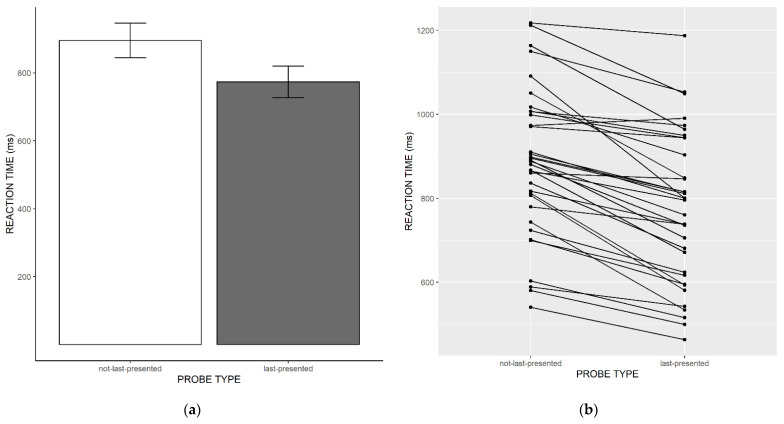
(**a**) Mean response times in ms for probes matching the last-presented item (“last-presented”) vs. probes matching other list items (“not-last-presented”). Error bars represent standard errors of the mean. (**b**) Individual mean response for probes matching the last-presented item (“last-presented”) vs. probes matching other list items (“not-last-presented”).

As can be seen in Figure 2a, and in line with what has previously been observed in children, our adolescent participants showed speeded responses to the last-presented memory item (mean = 774 ms, SD = 180), as compared to the not-last-presented memory item (mean = 884 ms, DS = 175). We assessed the evidence in the data for a last-presented benefit (i.e., faster responses for probes matching the last-presented item, compared to other target-presented probes, i.e., not-last-presented probes) with a paired, one-sided Bayesian *t*-test, and this revealed overwhelming evidence in favor of a last-presented benefit (BF = 2.31 × 10^8^). In other words, the data are 2.31 × 10^8^ times more likely under the alternative hypothesis according to which responses are faster for last-presented probes than for not-last-presented probes, than under the null hypothesis. This indicates that no spontaneous refreshing had occurred. This strongly suggests that adolescents did not switch away their attention from the last-encoded item. As can be seen in Figure 2b, this pattern appears to be very consistent across participants; almost all of them showed faster RTs for last-presented probes than for not-last-presented probes. Figures including the “new” probe condition as well as a one-way Bayesian ANOVA including the new probe condition can be found in the supplementary materials Figure S1.

## 4. Discussion

The current study is the first to use the last-presented benefit index in a sample of adolescents to test the spontaneous occurrence of attentional refreshing in 15-year-olds. Our results show that, overall, adolescents are faster to respond to a probe corresponding to the last-presented memory item than to a probe corresponding to another memory item. This observation is in contrast with the idea that adolescents might use spontaneous refreshing in an item-recognition task when they have enough time, as adults do (Vergauwe and Langerock 2017). Instead, our results indicate that the attentional focus of 15-year-olds remains on the last-encoded memory item, and thus, that 15-year-olds do not switch their attention away from the last-presented item to initiate refreshing. As such, the pattern of results observed for adolescents seems to deviate from what has been observed in young adults with less time available. Instead, the pattern of results observed for adolescents resembles the one that has been found in school-aged children (Vergauwe et al. 2021). Indeed, even when the to-be-remembered items were presented very slowly, at a rate of an item every 2500 ms, no evidence for spontaneous refreshing was found in 9- and 12-year-olds in Vergauwe et al. (2021). However, Vergauwe et al. (2021) have shown that 10-year-olds can switch attention away from the last-presented item when they are instructed to do so, even when the list is presented at a rate of 1 item every 1000 ms (Vergauwe et al. 2021, Experiment 2). Together, this suggests that children as young as 10 years of age are able to switch attention away from the last-encoded item with a faster item presentation than the one provided in the present paper, but that they do not do it spontaneously even when ample time is provided. Thus, even though the presentation rate used on the present study (1 item every 1500 ms) seems more than adequate to allow to 15-year-olds to refresh spontaneously, we did not observe any evidence for spontaneous refreshing and would not expect any difference in our results with 15-year-olds if an even slower presentation rate were to be used (e.g., 1 item every 2500 ms). 

The presence of the last-presented benefit in adolescents when using a rather slow presentation rate, as compared to the absence in adults with even less available time, suggests that adults spontaneously use some form of active maintenance strategy that is not mature yet in adolescents, which is in sharp contrast to the literature on the development of spontaneous refreshing (e.g., Barrouillet et al. 2009; Barrouillet and Camos 2020). This finding has at least two important implications: (1) attentional refreshing is not as general as previously thought (see also Vergauwe et al. 2021 for a similar argument), and (2) adolescents’ working memory performance is qualitatively different from adults’ working memory performance, and thus worth studying in more detail. These implications are discussed in more detail below.

### 4.1. Attentional Refreshing Is Not as General as Previously Thought

Different developmental models of working memory assume that attentional refreshing plays a role in the age-related increase in working memory capacity (Barrouillet and Camos 2020; Shimi and Scerif 2017). According to these models, children as young as 7 years old can use refreshing either spontaneously or prompted by a (spatial) cue, and the ability of using refreshing develops with age, thereby sustaining the development of working memory (Barrouillet et al. 2009; Camos and Barrouillet 2011; Shimi et al. 2014; Shimi and Scerif 2017). However, the results of the present study seem to suggest that adolescents keep their attention focused on the last-presented memory item, even 1500 ms after the onset of the last-presented memory item. Hence, adolescents do not seem to spontaneously switch attention away from this last item to engage in maintenance strategies that involve focusing attention on other items represented in working memory, at least not in our task. This suggests that, until 15 years of age, participants do not use attentional refreshing to reactivate the to-be-remembered items in an item-recognition task witg the features described in the present study.

The fact that we can find evidence for spontaneous refreshing in adolescents in certain studies (Barrouillet et al. 2009) but not in the present study might be explained by the possibility that attentional refreshing is used only in the cases in which adolescents are forced by the task to switch attention away from the last-presented memory item (see Rosselet-Jordan et al. 2022 for a similar argument). Indeed, refreshing in adolescents has been observed in a complex-span task, i.e., a task in which a secondary task forces the focus of attention to switch away from the last-encoded item (Barrouillet et al. 2009). However, in the item-recognition task like the one we have used here, there is no such secondary task. One could assume that refreshing only occurs in those task situations that require the focus of attention to switch back and forth between processing and memory materials.

Relatedly, Barrouillet et al. (2009) inferred the occurrence of refreshing in children from the observation of a cognitive load effect in this population. The cognitive load effect is the linear decrease in recall performance as a function of the proportion of time during which a concurrent processing task captures attention such that the decaying memory traces cannot be refreshed. However, alternative accounts of the cognitive load effect exists and some of these do not rely on the assumption that free time is used for attentional refreshing (Oberauer et al. 2012). Thus, it is also possible that attentional refreshing does not occur in either task. In any case, together, the findings on refreshing in adolescents appear to indicate that the use of refreshing may not be as general as previously thought.

It is important to note that, if refreshing is used only in tasks in which the cognitive load effect can be measured, then the use of refreshing appears to be rather limited, and probably too limited for refreshing to explain a large part of working memory development. In fact, the age-related increase in working memory capacity can been found in a multitude of tasks, regardless of whether the task induces attention to switch away from the maintained items (Brockmole and Logie 2013; Dempster 1981; Gathercole et al. 2004; Ottem et al. 2007; Simmering and Perone 2013; Wilson et al. 1987). The present study did not aim at directly assessing the relationship between the spontaneous use of attentional refreshing and working memory development. In the future, more specific studies are needed to investigate how the spontaneous use of refreshing is related to age-related differences in working memory capacity.

It is also important to point out that, while the presence of the last-presented benefit constitutes strong evidence against attentional refreshing (i.e., refreshing cannot occur if attention stays on the last to-be-remembered item), the disappearance of the last-presented benefit does not per se constitute univocal evidence in favor of refreshing. In fact, the absence of the last-presented benefit in adults for slow presentation rates might have been caused by attention-based mechanisms other than refreshing. For example, Valentini et al. (2022) propose that it could also be the result of the spontaneous occurrence of list-wide consolidation (Rhodes and Cowan 2018), elaboration (Bartsch et al. 2018; Jonker and MacLeod 2015) or chunking (Chen and Cowan 2005; Portrat et al. 2016; Thalmann et al. 2019). The use of any of these attention-based mechanisms during list presentation could explain the disappearance of the last-presented benefit. The fact that we did not observe this in adolescents indicates that they did not use attention-based maintenance mechanisms in our task. Overall, if we assume that the disappearance of last-presented benefit reflects refreshing, then our conclusion is one in terms of refreshing, namely that the ability to spontaneously use refreshing in an item-recognition task must develop between 15 and 20 years of age. However, if we assume that the disappearance reflects the occurrence of another attention-based maintenance mechanism, then our conclusion is more general, namely that the ability to spontaneously use attention for working memory maintenance must develop between 15 and 20 years of age.

Whatever process the disappearance of the last-presented benefit is reflecting, the current findings, together with those of Vergauwe and colleagues in young adults (Vergauwe and Langerock 2017) and children (Vergauwe et al. 2021), strongly indicate that there are still important developmental differences between adolescents and adults. This suggests that working memory in adolescents may function in a way that is qualitatively different from that in adults.

### 4.2. The Importance of Studying Working Memory in Adolescence

As mentioned in the introduction, the developmental literature on working memory has often assumed that working memory capacity reaches adult-like performance in early to mid-adolescence (Gathercole et al. 2004; Riggs et al. 2006; Wilson et al. 1987). However, life-span studies suggest that working memory performance keep increasing until later (Brockmole and Logie 2013; Luna et al. 2004; Sander et al. 2011; Swanson 1999). The present findings seem to indicate that working memory functioning is qualitatively different between 15-year-olds and young adults, thereby corroborating the notion that working memory continues to develop until early adulthood. Specifically, our study seems to suggest that attention may be used differently between 15-year-olds and young adults in an item-recognition task. Accordingly, studying the age gap between 15 years old and 20 years old in more detail might shed new light on the mechanisms that are still developing during adolescence and that will lead to the attainment of working memory peak performance around 20 years old. 

Recently, Isbell et al. (2015) made an important step in this direction as they tested working memory capacity in 13-, 16- and 23-year-olds using a change-detection task. They found a significant increase in working memory capacity between all successive age groups, demonstrating the working memory does not reach “adult-like” performance during adolescence, and not even at 16 years of age. Thus, working memory capacity seems to keep increasing substantially during adolescence, a result that has been shown with different materials and tasks (Andre et al. 2016; Kwon et al. 2002; Luciana et al. 2005; Sander et al. 2011).

Particularly interesting might be to investigate how the use of attention in working memory tasks changes throughout adolescence and to pinpoint the change in the use of attentional strategies. Regarding the development of attention, it has been shown that adolescents from 12 to 16 years of age suffer more from the presence of distracters in working memory task than adults, a finding that has been linked to the immaturity of the frontoparietal attentional networks that support working memory (Spronk et al. 2012). Moreover, studies have shown that, compared to young adults, adolescents have decreased activation on the inferior frontal cortex (Andre et al. 2016), which plays an important role in domain-general, rapid switching of attention (Ravizza et al. 2004).

### 4.3. Limitations of the Study

Finally, it has to be noted that while this study is a first, exploratory step towards understanding refreshing during adolescence, it has a few limitations. Firstly, the current study does not provide a direct comparison with adults using the same exact task as we used in adolescents. Indeed, the only comparison that can be made at this point is between conditions in which adults showed evidence for spontaneous refreshing when lists are presented such that there is enough time to use refreshing (presentation rate of 1 item per 1000 ms, in Vergauwe and Langerock 2017) and the current condition in which adolescents do not show evidence for spontaneous refreshing when lists are presented at a rate that is even slower than the rate used in adults (presentation rate of 1 item per 1500 ms in the current study). It is reasonable to assume that, if adults use spontaneous refreshing at a presentation rate of 1000 ms (but not at faster presentation rates), adults also use spontaneous refreshing at slower rates such as the one used here. Secondly, the current study does not include multiple age groups between 12-year-olds and young adults. Instead, as a first step, we only examined refreshing in 15-year-olds. In order to pinpoint more directly and more precisely when spontaneous refreshing emerges during adolescence, future studies could examine at what age the last-presented benefit disappears with slow presentation rates by comparing multiple age groups between 12-year-olds and young adults with the same paradigm. Thirdly, and finally, the current study includes only one specific presentation rate. Our reasoning was that, as a first step, the most informative condition is one in which there is time for refreshing to occur, i.e., a rate that is slow enough for refreshing to occur once encoding is finished (a period between 150 ms and 500 ms, depending on the memory item, is typically used as an appropriate encoding time for adolescents, see e.g., Isbell et al. 2015; Kwon et al. 2002). In order to obtain a complete picture of the developmental differences in the emergence of spontaneous refreshing, future studies that use the last-presented benefit to examine refreshing could include multiple presentation rates.

## 5. Conclusions

To conclude, the present paper shows that 15-year-olds display a last-presented benefit in a simple working memory task, which contrasts with the notion that adolescents use attentional refreshing to maintain information in working memory. Given that in young adults the last-presented benefit typically disappears when memory lists are presented more slowly, our results seem to suggest that working memory performance in adolescents is sustained by different mechanisms than in adults.

## Figures and Tables

**Table 1:** Studies supporting the occurrence of refreshing vs. a last-presented benefit in young children and adults.

Article	Exp	Presentation Rate (ms/item)	Participants’ Age Group	LPB Absent →Refreshing	LPB Present→ No Refreshing
(Valentini et al. 2022) *	1	350	Young adults (M = 21.03 years old)		X
(Valentini et al. 2022) *	2	350	Young adults (M = 20.42 years old)		X
(Vergauwe and Langerock 2017)	1	1000	Young adults (M = 21.13 years old)	X	
(Vergauwe and Langerock 2017)	2	500	Young adults (M = 21.45 years old)		X
(Vergauwe and Langerock 2017)	3	1000	Young adults (M = 21.61 years old)	X	
(Vergauwe and Langerock 2017)	4	350	Young adults (M = 21.60 years old)		X
(Vergauwe and Langerock 2017)	5	1000	Young adults (M = 23.03 years old)	X	
(Vergauwe et al. 2021)	1	500	9-year-olds		X
			12-year-olds		X
(Vergauwe et al. 2021)	1	1500	9-year-olds		X
			12-years-olds		X
(Vergauwe et al. 2021)	1	2500	9-years-olds		X
			12-years-olds		X

* Note that Valentini et al. (2022) manipulated the delay between item presentation and test probe, but of interest here is their 0 ms delay condition that replicates Experiment 4 in Vergauwe and Langerock (2017). Note: LPB stands for last-presented benefit.

## Data Availability

All materials and code can be found in https://osf.io/ay87u/.

## Notes

1 It has to be noted that this study does not include a direct comparison between adolescents and adults under the same task conditions. However, this does not preclude an across-study comparison between our adolescent sample and the extended adult literature we describe in the section related to the last-presented benefit.

2 Note that we initially meant to display this screen halfway through the trials. However, before the start of data collection we added additional trials per probe type condition in order to have as many trials as possible under 30 min of duration. When the trials were increased from 90 trials (30 per probe type) to 105 trials (35 per probe type), the number for the half experiment was not updated.

3 Additionally, we also calculated the proportion of the two types of errors participant could make (i.e., no response errors, i.e., trials in which participants did not respond within 3 s after the probe presentation, and incorrect responses, i.e., trials in which participants misjudged the probe) across the three levels of probe type. For last-presented probes, we observed 93% correct responses, 5% of incorrect response errors and 2% of no response errors. For not-last-presented probes, we observed 80% correct responses, 18% of incorrect response errors and 2% of no response errors. Finally, for new probes, we observed 88% correct responses, 10% of incorrect response errors and 2% of no response errors.

## Supplementary Materials

The additional analyses can be downloaded at: https://www.mdpi.com/article/10.3390/jintelligence11010004/s1, Figure S1: The “new” probe condition as well as a one-way Bayesian ANOVA including the new probe condition.
