# Supplementary material for: Stuck on the Last: The Last-Presented Benefit as an Index of Attentional Refreshing in Adolescents
Source: J Intell. 2022 Dec 23;11(1):4. doi: 10.3390/jintelligence11010004 (PMC9860735; doi:10.3390/jintelligence11010004)
Supplement: Supplementary file 1 [file jintelligence-11-00004-s001.zip › jintelligence-1957900-supplementary.pdf]

Supplementary Files

In addition to the analysis reported in the main text, we also ran a one-way Bayesian ANOVA on the reaction times, with Probe Type (3 levels: last-presented, not-last-presented, and new) as a repeated measure. The analysis resulted in a Bayes Factor of 66.23 in favour of the inclusion of the main effect of Probe Type. As can be seen in the Figure S1, the probes corresponding to the not-last-presented items (mean=884, SD= 175) and the "new" items (mean= 951 ms, SD= 195) were responded to more slowly than the probes corresponding to the last-presented items (mean= 774 ms, SD=180).

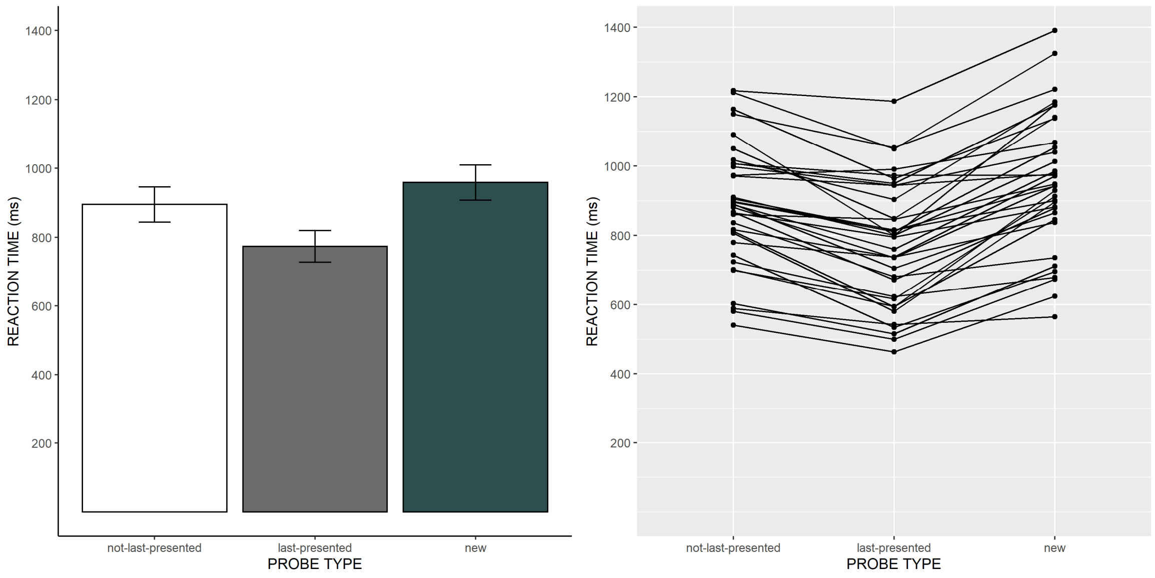

(a) **(b)**

Figure S1. (a) Mean response times in ms for probes matching the last-presented item ("last-presented"), probes matching other list items ("not-last-presented"), and probes not matching any presented item ("new"). Error bars represent standard errors of the mean; (b) Individual mean response for probes matching the last-presented item ("last-presented"), probes matching other list items ("not-last-presented") and probes not matching any presented item ("new").
